# Therapeutic Regulation of Macrophage Polarization for Diabetic Kidney Disease by Targeted Metabolic Reprogramming

**DOI:** 10.1007/s11596-026-00192-x

**Published:** 2026-04-17

**Authors:** Si-ying Fei, Rui-tong Liu, Wen-xiao Yang, Bing-shu Wu, Xiao-bin Mei, Mao-jin Xu

**Affiliations:** 1 School of Basic Medical Sciences, Naval Medical University, Shanghai, 200433 China; 2 Department of Geriatrics, The First Affiliated Hospital of Naval Medical University, Shanghai, 200433 China; 3 Department of Internal Medicine Teaching and Research Office, The First Affiliated Hospital of Naval Medical University, Shanghai, 200433 China; 4 Department of Nephrology, The First Affiliated Hospital of Naval Medical University, Shanghai, 200433 China

**Keywords:** Metabolic reprogramming, Macrophage polarization, Macrophage phenotype, Diabetic kidney disease, Renal fibrosis, Mitochondrial function, Diabetes complications

## Abstract

Diabetic kidney disease (DKD) is one of the most common microvascular complications of diabetes. It can be identified by thickening of the glomerular basement membrane, reduced glomerular filtration rate, and persistent proteinuria. Macrophages play a key role in the pathogenesis of DKD, and their phenotype (M1 and M2) is finely regulated by metabolic reprogramming. M1 macrophages exacerbate inflammatory damage and fibrosis in renal tissue by secreting pro-inflammatory mediators and reactive oxygen species (ROS). M2 macrophages (further subdivided into M2a, M2b, M2c and M2d subtypes) primarily exert anti-inflammatory and tissue-repairing effects. Of these, the M2a and M2c subtypes are particularly crucial for anti-inflammatory repair. This study aimed to systematically review the mechanisms by which glucose, lipid, amino acid, and mitochondrial function-related metabolism influence macrophage polarization. It further explored therapeutic strategies to mitigate renal inflammation and fibrosis by regulating macrophage polarization through targeted metabolic pathways, including inhibiting glycolysis, promoting fatty acid oxidation, modulating amino acid metabolism, and enhancing mitochondrial biogenesis and oxidative phosphorylation (OXPHOS). Several natural compounds and synthetic drugs exhibit the potential to induce M2 polarization and suppress M1 polarization through metabolic reprogramming, thereby offering new directions for optimizing therapeutic strategies for DKD.

## Introduction

Diabetic kidney disease (DKD) is characterized by persistent proteinuria, a progressive decline in the estimated glomerular filtration rate (eGFR), and thickening of the glomerular basement membrane. As one of the most severe complications of diabetes, it affects approximately 40% of patients with type 2 diabetes (T2D) and 30% of those with type 1 diabetes (T1D) [1]. Epidemiological studies indicate that DKD is the leading cause of end-stage renal disease (ESRD) globally [2]. Current clinical approaches to glycemic control and lipid management for this disease have not yielded positive outcomes. Other mechanisms underlying metabolic disorders that lead to renal damage require further investigation to identify effective therapies [3].

Macrophages are pivotal in the pathogenesis of kidney disease and represent potential therapeutic targets for renal injury and fibrosis [4]. Single-cell RNA sequencing (scRNA-seq) revealed that renal macrophages are the predominant infiltrating immune cells in early-stage DKD. Renal macrophages can be polarized into pro-inflammatory M1-like or anti-inflammatory M2-like phenotypes under the influence of various signaling molecules, thereby exacerbating renal inflammation or promoting renal repair. In DKD, the polarization of renal macrophages influences disease progression through multiple mechanisms. M1 macrophages exacerbate inflammation and tissue damage by producing pro-inflammatory mediators and reactive oxygen species (ROS), whereas M2 macrophages possess anti-inflammatory and reparative properties. Hyperglycemia and oxidative stress can induce M1 polarization, promoting renal fibrosis and worsening kidney function. Furthermore, macrophage polarization influences interactions between renal cells, further driving DKD progression [5].

Metabolic reprogramming is a mechanism by which cells alter their metabolic patterns to match the energy demands of cell survival and growth. It can regulate macrophage polarization in diabetes. Therefore, targeting metabolic reprogramming in renal macrophages of patients with DKD to alter the polarization balance may hold promise for improving outcomes in patients with diabetes and preventing or delaying the progression of DKD to ESRD, thereby potentially overcoming current therapeutic challenges in DKD management [6].

## Role of Various Macrophage Phenotypes in DKD

**Fig. 1:**
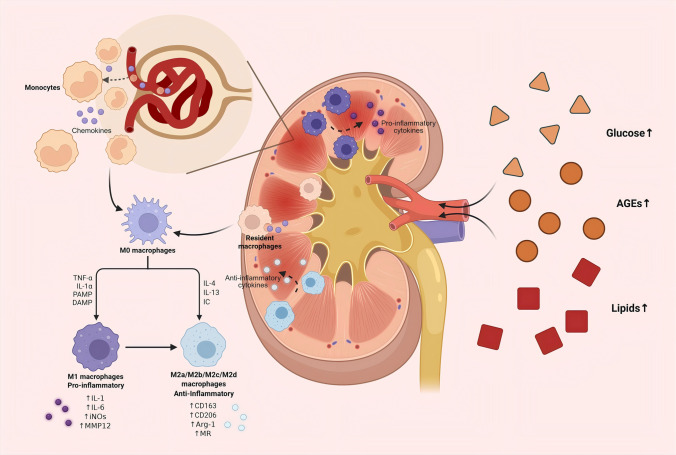
Macrophage polarization within the kidneys in DKD. Elevated levels of glucose, AGEs, lipids, and other diabetic factors promote the expression of cytokines by renal parenchymal cells, leading to the transformation of kidney-resident macrophages into M0 macrophages. Concurrently, circulating monocytes are recruited to the kidney via cytokine-mediated chemotaxis and differentiate into M0 macrophages. Under stimulation by TNF-α, IL-1α, PAMPs, and DAMPs, M0 macrophages polarize into M1 pro-inflammatory macrophages, characterized by upregulated expression of IL-1, IL-6, iNOS, and MMP12, thereby contributing to renal inflammatory responses. Conversely, in the presence of IL-4, IL-13, and ICs, M0 macrophages polarize into anti-inflammatory M2 subtypes (M2a, M2b, M2c, and M2d), resulting in increased expression of CD163, CD206, Arg-1, and MR and the ability to exert anti-inflammatory effects to facilitate renal repair. *AGEs,* advanced glycation end products; *TNF-α,* tumor necrosis factor-alpha; *IL-1α,* interleukin-1 alpha; *PAMPs,* pathogen-associated molecular patterns; *DAMPs,* damage-associated molecular patterns; *iNOS,* inducible nitric oxide synthase; *MMP12,* matrix metalloproteinase-12; *IC,* immune complexes; *Arg-1,* arginase-1; *MR,* mannose receptor

Macrophages are critical in DKD. Different macrophage phenotypes exhibit distinct functions in the kidney. On the basis of their origin, macrophages can be classified into tissue-resident macrophages and monocyte-derived macrophages [7] (Fig. 1). Kidney-resident macrophages (KRMs) can be derived from yolk sac erythro-myeloid progenitors, fetal liver monocytes, or hematopoietic stem cells (HSCs). Macrophages in adult tissues primarily originate from the yolk sac or fetal liver during embryonic development [8]. KRMs exhibit diverse functions in different stages of kidney disease. They support renal repair in acute kidney injury (AKI) by promoting angiogenesis, creating an anti-inflammatory environment, and demonstrating proliferative capacity during repair. However, in chronic kidney disease (CKD), KRMs may participate in the progression of renal fibrosis during ESRD, thus exacerbating this condition through the production of pro-inflammatory cytokines. Moreover, these cells demonstrate protective effects under certain circumstances [4]. Research confirms that renal injury induces the local proliferation and differentiation of resident macrophages into repair-promoting macrophages, facilitating repair through mechanisms such as upregulating Vegfa expression [9]. Another study indicated that ROS produced by resident macrophages during the DKD process may exacerbate damage, while their phagocytic function contributes to tissue repair [7]. These findings suggest that KRMs play a dual role in DKD.

Another small fraction of macrophages in the kidney originates from the bone marrow. When renal macrophage niches are disrupted, monocytes differentiated from HSCs migrate to the kidney under the influence of chemokines, where they differentiate into mature macrophages and perform specific functions [10]. ScRNA-seq trajectory analysis revealed that extracellular matrix remodeling–associated macrophages derived from monocytes were polarized toward M1 macrophages after renal injury. In addition to extracellular matrix–associated components and proteases, these macrophages exhibited high expression of genes related to lipid metabolism and phagocytosis, with both ECM and lipid metabolism reprogramming jointly promoting renal fibrosis [9]. In DKD, high concentrations of glucose and advanced glycation end products (AGEs) promote the expression of intercellular adhesion molecule-1 on renal parenchymal cells (RPCs), thereby facilitating the recruitment of circulating monocytes. Concurrently, RPCs and KRMs express chemotactic factors that induce the differentiation of circulating monocytes into infiltrating macrophages, ultimately leading to renal injury [11].

Macrophages can be classified into two major subsets on the basis of their activation state and function: M1 (also known as the pro-inflammatory phenotype) macrophages and M2 (also known as the anti-inflammatory phenotype) macrophages [12]. Upon cytokine stimulation, monocyte-derived macrophages can readily differentiate into M1 or M2 cells. In contrast, KRMs do not produce arginase or nitric oxide (NO) when stimulated by cytokines and transform into a macrophage population that exhibits hybrid phenotypes during renal inflammation [13]. These macrophages infiltrate renal tissue and release various inflammatory mediators and cytokines. This induces inflammatory responses and oxidative stress, leading to tissue damage and fibrosis. The infiltration of both M1 and M2 macrophages is also observed in the kidneys of patients with DKD [5].

M1 macrophages can express pro-inflammatory factors, including interleukin (IL)-1, IL-6, inducible nitric oxide synthase (iNOS), and matrix metalloproteinase-12 (MMP12), upon induction by pathogen-associated molecular patterns, danger-associated molecular patterns, and pro-inflammatory cytokines such as IL-1α and TNF-α, leading to tissue inflammation and damage. M2 macrophages can be derived from infiltrating monocytes or transformed from M1 macrophages under various stimuli involving IL-4, IL-13, or immune complexes (ICs). They suppress inflammatory responses and promote tissue repair through high expression of CD206, CD163, arginase-1 (Arg-1), and mannose receptor (MR) [11, 12]. M2 macrophages can be further classified into various subpopulations: M2a, M2b, M2c, and M2d. In DKD, different subtypes exhibit distinct functions. M2a cells participate in promoting wound healing and tissue fibrosis, and M2b cells are involved in immune regulation, whereas M2c cells are associated with immunosuppression, matrix remodeling, and tissue repair [12]. M2c cells combat renal inflammation and fibrosis through the secretion of IL-10/MMP7. Targeting the induction of M2c polarization offers a novel therapeutic approach to simultaneously alleviate inflammation and fibrosis [14]. M2d cells are more frequently associated with tumors. Further exploration of its role in DKD is anticipated. Although macrophages can transform into M2 macrophages for tissue repair, prolonged exposure to hyperglycemia compromises the anti-inflammatory repair properties of M2 macrophages. This leads to a dysregulated state characterized by the coexistence and competition of macrophage phenotypes, resulting in disease progression to ESRD [12].

## Relationship Between Macrophage Phenotype and Metabolism

### Glucose Metabolism

#### Glycolysis

**Fig. 2:**
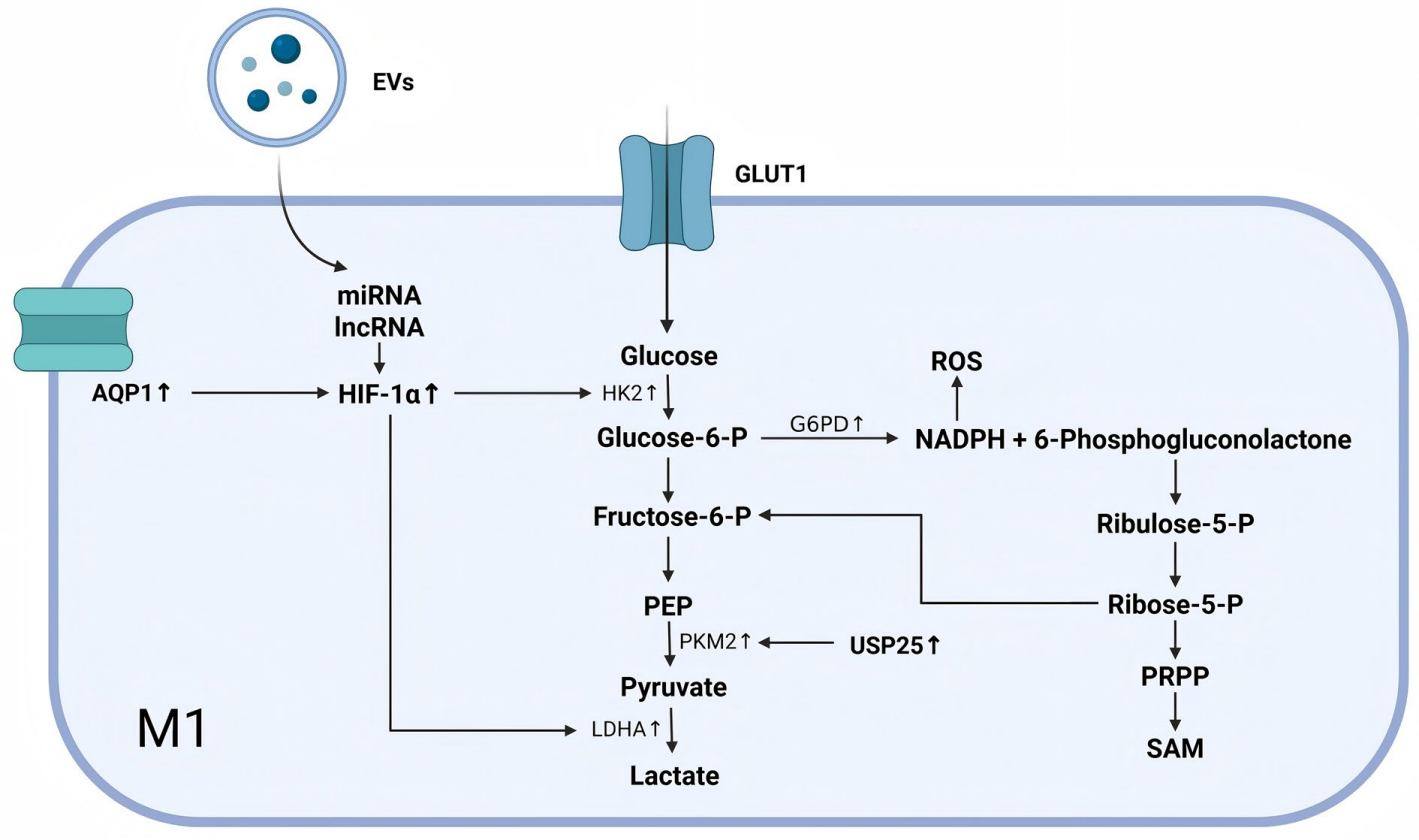
Glucose metabolism in M1 macrophages. *EVs,* extracellular vesicles; *GLUT1,* glucose transporter 1; *AQP1,* aquaporin 1; *HIF-1α,* hypoxia-inducible factor-1 alpha; *HK2,* hexokinase 2; *G6PD,* glucose-6-phosphate dehydrogenase; *ROS,* reactive oxygen species; *PKM2,* pyruvate kinase M2; *USP25,* ubiquitin-specific protease 25; *LDHA,* lactate dehydrogenase A; *PRPP,* phosphoribosyl pyrophosphate; *SAM,* S-adenosylmethionine

Glycolysis is the initial step in glucose metabolism. Glucose is transported into cells via GLUT1 and phosphorylated by hexokinase (HK2). It subsequently breaks down into pyruvate or lactate under anaerobic conditions. Its metabolic intermediates also serve as precursors for metabolic pathways [15]. Extracellular vesicles (EVs) released by renal tubular epithelial cells carry miRNAs and lncRNAs in high-glucose environments. These EVs increase their stability by inhibiting HIF-1α hydroxylation and activating key glycolytic enzymes (e.g., HK2 and LDHA) to drive glycolysis. This process not only supplies energy to macrophages but also directly promotes inflammatory cytokine expression through metabolic byproducts (e.g., lactate), thereby promoting macrophage polarization toward the M1 phenotype [16] (Fig. 2). Additionally, USP25 and AQP1 enhance glycolytic metabolism by regulating the activity of glycolytic enzymes (e.g., PKM2) and HIF-1α expression, thereby driving the release of pro-inflammatory cytokines and promoting M1 polarization [17, 18]. Glycolysis, as a pivotal hub in energy metabolism and inflammatory regulation, primarily drives M1 polarization. It exacerbates the progression of DKD through a cascade of inflammatory mediators, such as IL-6 and TNF-α.

However, lactate, a metabolic product of glycolysis, may also function as a signaling molecule to regulate M2 polarization in macrophages. Elevated PKM2 activity in renal tubular cells promotes lactate production, which upregulates TGF-β1 transcription by inducing histone H3K18 lactylation. Secreted TGF-β1 activates the Smad3 pathway in macrophages, leading to their M2 polarization. Puerarin inhibits PKM2 to block this pathway and reduce fibrosis [19]. This mechanism also provides a theoretical basis for targeting the glycolysis‒lactic acid axis in the treatment of DKD.

#### Pentose Phosphate Pathway (PPP)

The PPP is divided into oxidative and nonoxidative branches: the oxidative branch (oxPPP) generates NADPH, whereas the nonoxidative branch (nonoxPPP) reassembles the carbon skeleton. The core function of the pathway lies in supplying NADPH and pentoses, which are crucial for cellular redox homeostasis, immune responses, and biosynthesis [20]. The PPP plays multiple roles in the M1 polarization of macrophages through dynamic crosstalk between oxPPP and nonoxPPP. First, oxPPP catalyzes NADPH production via glucose-6-phosphate dehydrogenase (G6PD), thus maintaining the intracellular redox balance and driving substantial ROS generation. ROS not only directly participate in pathogen clearance but also activate pro-inflammatory signaling pathways such as the NF-κB and p38 MAPK pathways, thereby forming a positive feedback loop between oxidative stress and inflammation [21]. For example, in a DIO mouse model, the upregulation of GLUT1 expression in adipose tissue macrophages led to increased glucose uptake and NADPH levels via the oxPPP pathway. This subsequently exacerbates ROS-dependent inflammatory responses, promoting insulin resistance and metabolic disorders [22]. Second, ribose-5-phosphate generated by the PPP can synthesize phosphoribosyl pyrophosphate, providing precursors for nucleotide synthesis and supporting the generation of S-adenosylmethionine (SAM). As a methyl donor, SAM is used to mediate histone methylation (e.g., H3K4me3), directly driving the transcription of inflammatory cytokines such as IL-1β and TNF-α [21]. Inhibiting G6PD or oxPPP activity significantly reduces ROS levels and pro-inflammatory cytokine secretion and inhibits M1 polarization [22]. These multilayered metabolic regulatory mechanisms collectively establish the pivotal role of the PPP in M1 polarization, thereby offering potential therapeutic approaches for chronic inflammation and metabolic diseases by targeting immune metabolism, such as by inhibiting G6PD or modulating ROS signaling pathways.

### Lipid Metabolism

#### Fatty Acids

Fatty acid metabolism is a core process in cellular energy supply. Free fatty acids (FFAs) are activated by acyl-CoA and mitochondrial transport mediated by CPT. They are then cleaved via β-oxidation into acetyl-CoA, generating FADH₂/NADH to drive ATP synthesis. Excess acetyl-CoA is esterified into triglycerides and stored in lipid droplets for energy reserve [23]. In DKD, macrophages with varying phenotypes exhibit distinct fatty acid metabolic characteristics, leading to different functions.

**Fig. 3:**
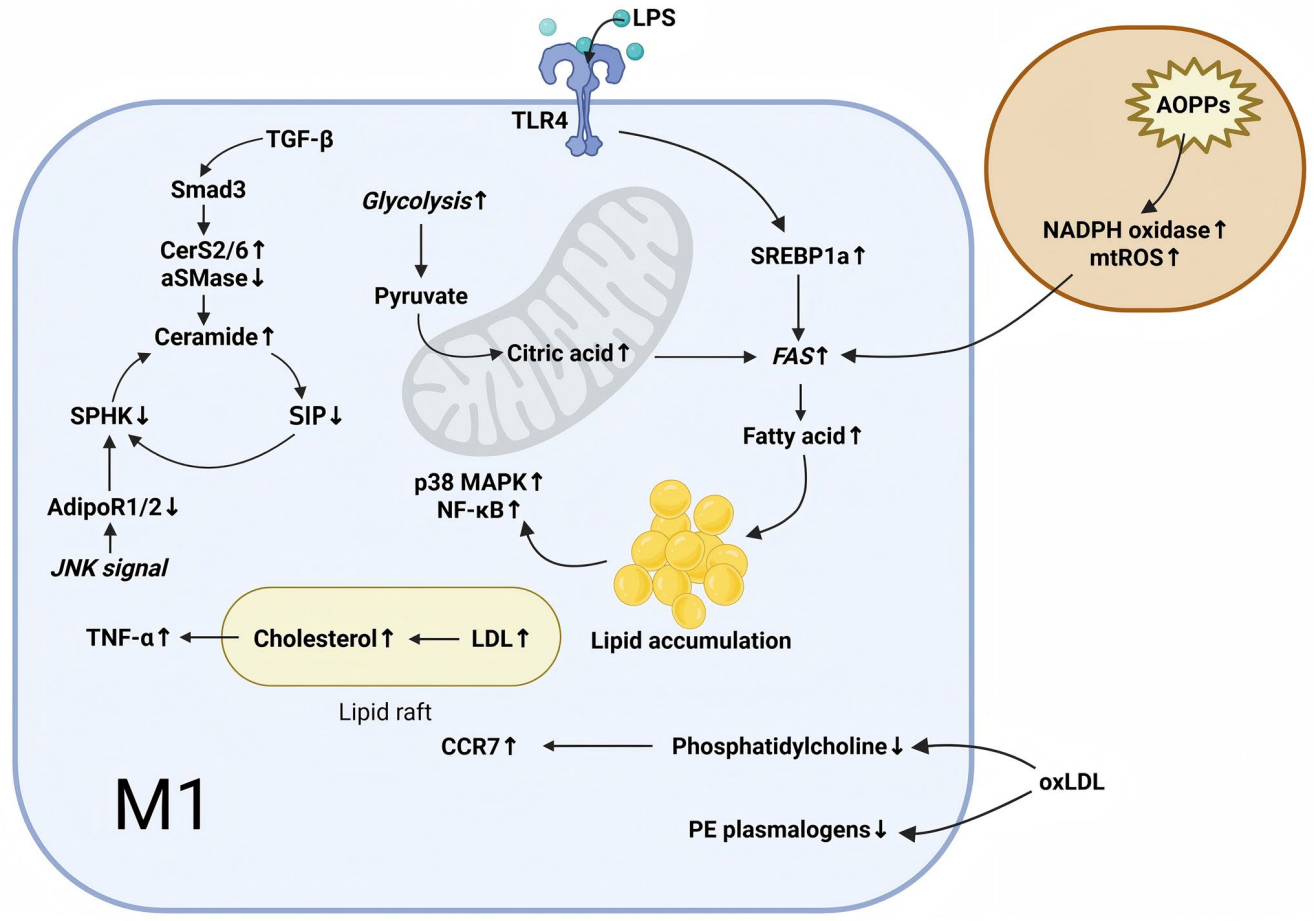
Lipid metabolism in M1 macrophages. *LPS,* lipopolysaccharide; *TLR4,* toll-like receptor 4; *SREBP1a,* sterol regulatory element-binding protein 1a; *FAS,* fatty acid synthesis; *AOPPs,* advanced oxidation protein products; *mtROS,* mitochondrial reactive oxygen species; *oxLDL,* oxidized low density lipoprotein; *CCR7,* C–C chemokine receptor type 7; *AdipoR1/2,* adiponectin receptor ½; *SPHK,* sphingosine kinase; Smad3, SMAD family member 3; *CerS2/6,* ceramide synthase 2/6; *aSMase,* acid sphingomyelinase; *S1P,* sphingosine-1-phosphate

Fatty acid synthesis (FAS) is a key metabolic step for M1 macrophage function [24] (Fig. 3). In a high-glucose environment, glycolysis results in the production of pyruvate, which enters mitochondria and is transformed into citrate. This citrate participates in regulating FAS activity, promotes inflammation and fibrosis, and mediates lipid accumulation, ultimately leading to kidney injury [25]. High-glucose environments significantly upregulate the activity of key enzymes in FAS (e.g., FASN and ACC), leading to excessive lipid accumulation in macrophages and triggering endoplasmic reticulum stress. This subsequently activates pro-inflammatory pathways, including p38 MAPK and NF-κB, driving the release of M1-type pro-inflammatory cytokines such as TNF-α and IL-1β, thereby amplifying local inflammatory responses [24]. Similarly, stimuli such as LPS activate abnormal FAS through the TLR4/SREBP1a axis, exacerbating M1 polarization and inflammatory responses [26]. Additionally, during ESRD, advanced oxidation protein products increase lipolysis by activating adipocyte NADPH oxidase and mtROS, thereby releasing FFAs. These FFAs simultaneously promote macrophage infiltration via the NF-κB/MCP-1 axis while directly inducing the upregulation of M1 macrophage markers (e.g., iNOS) and the downregulation of anti-inflammatory markers (e.g., CD206), thereby forming a vicious cycle of inflammation that accelerates renal lipoatrophy [27]. These findings provide new insights for intervention strategies targeting FAS metabolism in the treatment of DKD.

**Fig. 4:**
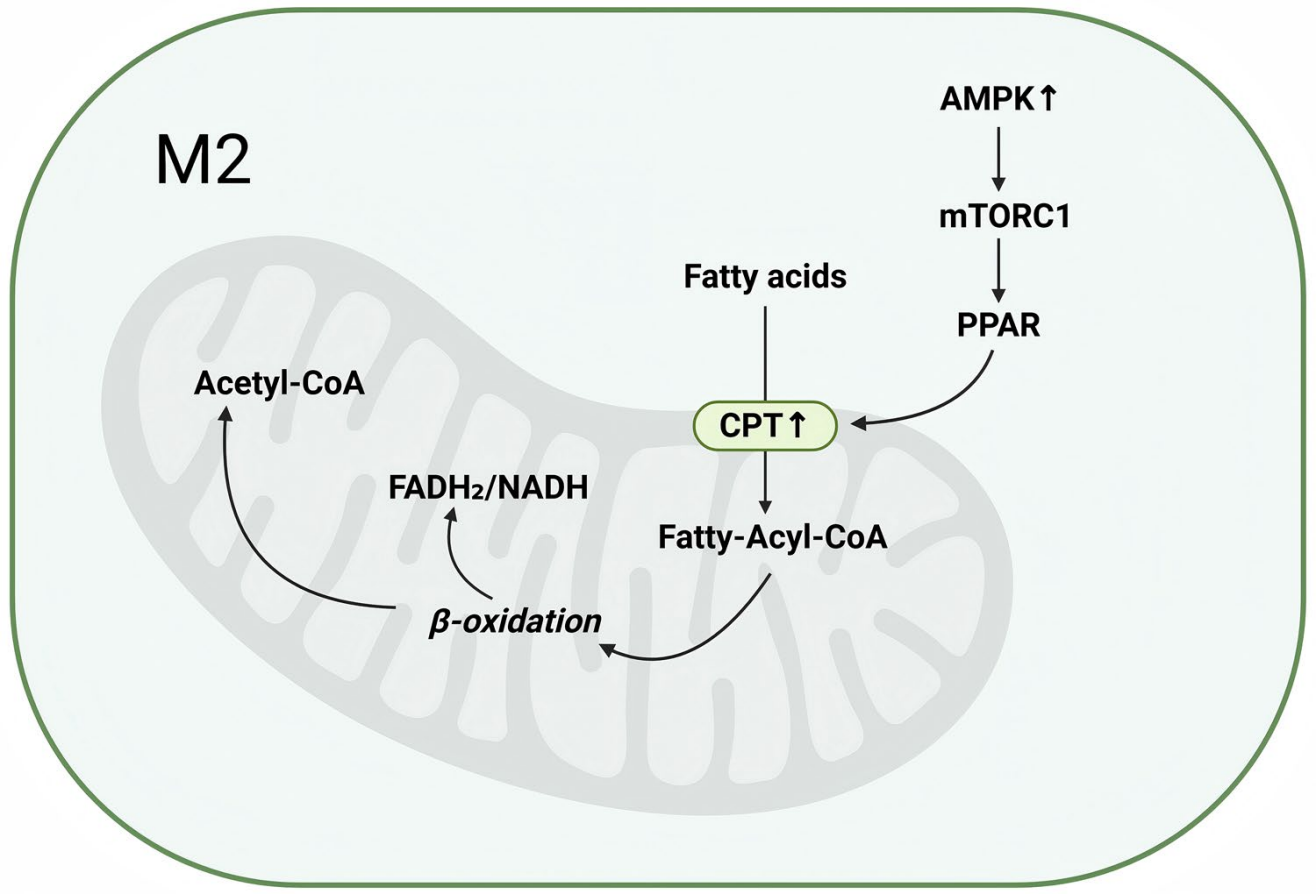
Lipid metabolism in M2 macrophages. *AMPK,* AMP-activated protein kinase; *mTORC1,* mechanistic target of rapamycin complex 1; *CPT,* carnitine palmitoyltransferase

Unlike M1 macrophages, M2 macrophages primarily rely on fatty acid oxidation (FAO) to maintain their function (Fig. 4). AMPK activation suppresses mTORC1 activity, promotes FAO, and inhibits FAS activity, thereby reducing the formation of a lipotoxic microenvironment. Additionally, PPARα agonists (e.g., fenofibrate) increase the expression of FAO-related genes such as *CPT1*, directly increasing fatty acid breakdown capacity and alleviating lipid accumulation [24]. Soluble uric acid (sUA) has been demonstrated to increase macrophage FAO, upregulate the expression of genes such as *CPT1* and *PPARγ*, and reduce the release of ROS and pro-inflammatory factors in renal diseases. Concurrently, it activates the PPARγ–PGC1β axis to drive the expression of M2 markers, such as Arg-1, and inhibits iNOS, thereby inducing M2 polarization and alleviating renal injury [28].

#### Phosphatidylcholine (PC)

PC and its metabolites can regulate macrophage polarization through multiple pathways. Oxidized low-density lipoprotein (oxLDL) reduces the membrane PC/free cholesterol ratio, disrupts membrane fluidity, inhibits SREBP2 target genes (e.g., *LDLR* and *FASN*), and upregulates the M1 marker CCR7. Concurrently, oxLDL induces a reduction in arachidonic acid (AA)-containing phosphatidylethanolamine (PE) plasmalogens and impairs their antioxidant function, collectively driving macrophage polarization toward the M1 phenotype [29]. Disrupted sphingolipid metabolism, leading to ceramide accumulation, also plays a pivotal role in regulating polarization. High-glucose environments upregulate ceramide synthase (CerS2/6) expression by activating the TGF-β/Smad3 pathway while simultaneously suppressing acid sphingomyelinase (aSMase) activity, resulting in enhanced ceramide synthesis and impaired degradation. Insulin resistance inhibits the expression of the adipokine receptor AdipoR1/2 via the JNK pathway, reducing sphingosine kinase (SPHK) activity and further blocking the process of converting ceramides into sphingosine-1-phosphate (S1P) [24]. Long-chain ceramides drive the release of pro-inflammatory factors (IL-6 and TNF) during M1 polarization by enhancing TLR4 signaling and activating NF-κB. In contrast, their downstream metabolites (e.g., glycosphingolipids and gangliosides) promote macrophage polarization toward the M2 phenotype by inhibiting TLR4 membrane translocation or interfering with MyD88 binding during the inflammation resolution phase [30]. Additionally, S1P, the metabolic end product of ceramides, can promote inflammatory responses through sphingosine kinase 2 (Sphk2). S1P can upregulate Sphk2 activity, thereby maintaining pro-inflammatory metabolic characteristics by enhancing glycolytic activity and promoting the expression of M1 macrophage cytokines such as TNF-α and IL-1β [31].

#### Cholesterol

Cholesterol metabolism maintains dynamic equilibrium under normal physiological conditions through three pathways: the synthetic pathway is regulated by SREBP to mediate intracellular synthesis, the inflow pathway is mediated by LDL receptors to mediate endocytosis, and the efflux pathway is facilitated by transporters such as ABCA1. Additionally, free cholesterol in the endoplasmic reticulum can be esterified by sterol O-acyltransferase 1 (SOAT1/ACAT1) and stored in lipid droplets, collectively maintaining cholesterol homeostasis [32]. Dysregulation of renal cholesterol metabolism is a key driver in the pathogenesis of DKD [23]. Recent studies have indicated that the Bloch and Kandutsch–Russell pathways in diabetic monocytes/macrophages are significantly activated, leading to abnormal accumulation of the intermediate desmosterol. This induces an atypical polarization state in which cells simultaneously express cytokines associated with both M1 and M2 macrophages, thereby damaging the glomerular filtration barrier through direct contact or the secretion of mediators [33]. Additionally, cholesterol metabolism can directly influence the direction of polarization by regulating the composition of lipid rafts in macrophage membranes. Hypercholesterolemia increases LDL influx into lipid rafts, thus increasing their cholesterol content. It also drives macrophage polarization toward the M1 phenotype by enhancing inflammatory signaling, such as TNF-α signaling. In contrast, statins promote M2 polarization through mechanisms such as the inhibition of cholesterol synthesis [34]. At the genetic level, the APOL1 kidney risk variant (G1/G2) inhibits cholesterol efflux by downregulating the expression of the cholesterol efflux transporter ABCA1/ABCG1. This leads to M1 macrophage formation and the release of inflammatory mediators, thereby exacerbating renal injury [35].

### Amino acid Metabolism

#### Arginine

**Fig. 5:**
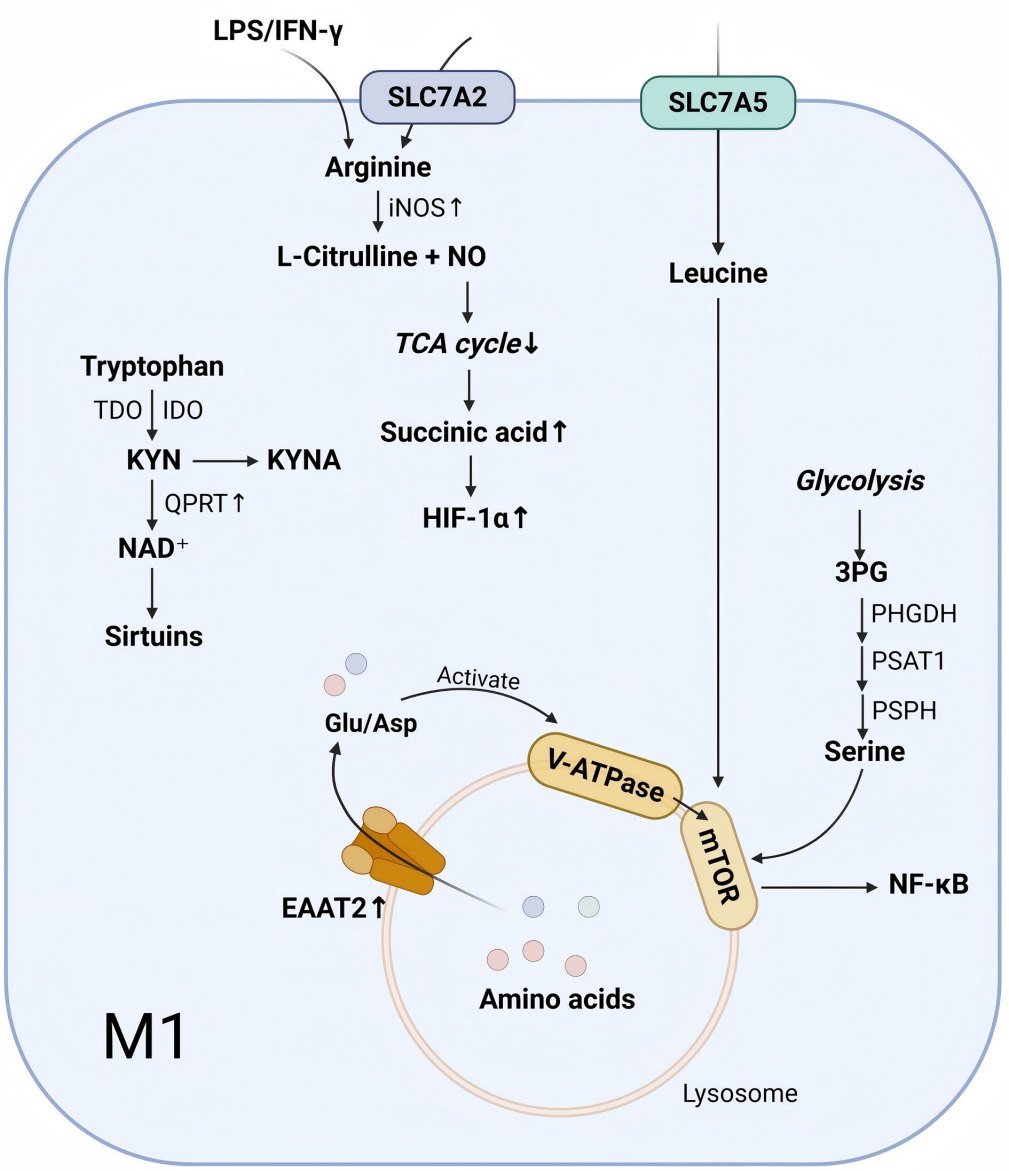
Amino acid metabolism in M1 macrophages. *TDO,* tryptophan 2,3-dioxygenase; *IDO,* indoleamine 2,3-dioxygenase; *KYN,* kynurenine; *KYNA,* kynurenic acid; *QPRT,* quinolinic acid phosphoribosyltransferase; Glu, glutamate; *Asp,* aspartate; *EAAT2,* excitatory amino acid transporter 2; *3PG,* 3-phosphoglycerate; *PHGDH,* phosphoglycerate dehydrogenase; *PSAT1,* phosphoserine aminotransferase 1; *PSPH,* phosphoserine phosphatase; *mTOR,* mammalian target of rapamycin

**Fig. 6:**
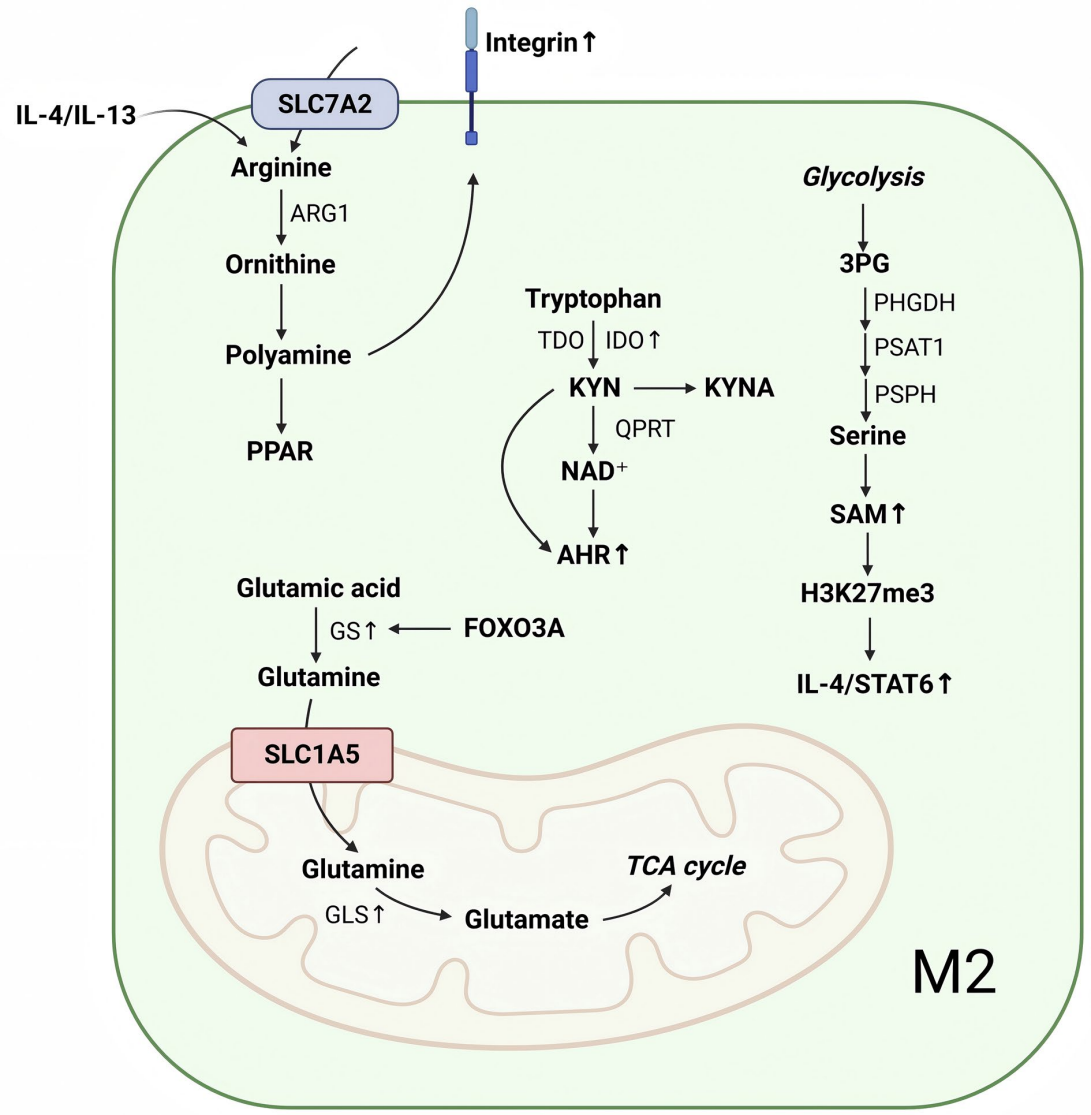
Amino acid metabolism in M2 macrophages. *ARG1,* arginase 1 *PPAR,* peroxisome proliferator-activated receptor; *GS,* glutamine synthetase; *GLS,* glutaminase; *AHR,* aryl hydrocarbon receptor

Arginine metabolism plays a crucial role in macrophage polarization through multiple pathways. After LPS/IFN-γ stimulation, M1 macrophages highly express iNOS, which catalyzes the conversion of arginine to NO. This activity inhibits the tricarboxylic acid cycle and induces succinate accumulation, thereby activating the HIF-1α/IL-1β axis to promote inflammatory responses [36]. iNOS-mediated NO production also serves as the key molecular basis for the phagocytic function of M1 macrophages [37] (Fig. 5). In contrast, M2 macrophages rely on Arg-1 to convert arginine into ornithine and polyamines (putamine/spermine) under the influence of IL-4/IL-13 (Fig. 6). Polyamines increase mitochondrial translation efficiency and regulate integrin expression to promote the phagocytosis of apoptotic cells. They also synergize with arginine methyltransferase–mediated epigenetic regulation to stabilize M2 phenotype–associated transcription factors (e.g., PPARγ) [36, 38]. Multiple studies have confirmed that imbalances in arginine metabolism directly influence macrophage phenotypes and their functions. For instance, DsbA-L gene deletion downregulates iNOS expression, reduces the proportion of M1 macrophages, and increases M2 macrophage infiltration in DKD-associated models, thereby mitigating inflammatory damage in renal tissue [39]. However, the inhibition of iNOS-induced arginine accumulation also exacerbates renal injury by promoting ROS accumulation and pro-inflammatory cytokine release in salt-sensitive hypertension models, thereby driving macrophage polarization toward the M1 phenotype [40]. These findings collectively elucidate how arginine metabolism regulates macrophage polarization through multiple pathways, thereby offering new strategies for targeted intervention in inflammatory kidney injury.

#### Tryptophan

Tryptophan is primarily metabolized by the kynurenine pathway (KP) under physiological conditions, with the rate-limiting enzymes indoleamine 2,3-dioxygenase (IDO) and tryptophan 2,3-dioxygenase catalyzing this process. IDO can be activated by inflammatory cytokines such as TNF-α, IL-6, and IFN-γ. This protein catalyzes the formation of kynurenine (KYN), which is further metabolized into neuroprotective kynurenic acid and nicotinamide adenine dinucleotide (NAD⁺), among other products [41]. In DKD, disrupted tryptophan metabolism primarily manifests as abnormal activation of KP, leading to the accumulation of toxic metabolites such as KYN and the depletion of protective metabolites, resulting in renal injury [42].

Disrupted tryptophan metabolism can influence the progression of DKD by regulating macrophage polarization. Inflammatory cytokines such as IFN-γ induce high expression of IDO1, enhance KP metabolism, increase the levels of metabolites such as KYN, activate the aryl hydrocarbon receptor (AHR) signaling pathway, suppress TNF-α/IL-6 release, and promote IL-10 secretion, thereby driving M2 polarization of macrophages [43, 44]. M1 polarization relies on quinolinate phosphoribosyltransferase to accelerate NAD⁺ synthesis through its high expression. NAD⁺ acts as a cofactor for sirtuin deacetylases, thus maintaining pro-inflammatory gene expression via epigenetic mechanisms [43]. On the basis of the aforementioned mechanisms, targeting tryptophan metabolism to regulate macrophage polarization has therapeutic potential for DKD. These findings indicate that the Jianpi–Yishen formula significantly increases tryptophan metabolism levels, promotes KYN production, and activates AHR signaling pathway. This subsequently inhibits pro-inflammatory signaling pathways such as NF-κB, reduces the expression of M1 markers (e.g., CD86), and suppresses pro-inflammatory cytokine release, ultimately mitigating renal inflammatory injury and fibrosis [45].

#### Serine

Serine metabolism relies primarily on the serine synthesis pathway, which uses 3-phosphoglycerate, an intermediate product of glycolysis, through three enzymatic reactions catalyzed by PHGDH, PSAT1, and PSPH. This pathway is finely regulated by negative feedback from PKM2 and transcription factors such as ATF4 [46]. In DKD, hyperglycemia relieves the inhibition of the JNK pathway by downregulating dual specificity phosphatase 4 (DUSP4) in podocytes, leading to insulin resistance through IRS1 serine 307 phosphorylation. Concurrently, DUSP4 deficiency upregulates Nox4 expression and increases ROS production, ultimately causing podocyte injury and renal dysfunction [47]. Additionally, accumulated FFAs activate PKCα, induce phosphorylation of serine 313 (S313) in PACSIN2, and form a positive feedback loop with N-WASP. This ultimately disrupts the cytoskeletal reorganization capacity of podocytes, further leading to filtration barrier dysfunction [48]. These serine-related pathways collectively shape the inflammatory microenvironment of DKD.

Serine metabolism also plays a crucial role in macrophage polarization. It is key to maintaining the anti-inflammatory activity and proliferation of M2 macrophages; its inhibition promotes the polarization of M2 to M1 macrophages [49]. Mechanistic studies indicate that inhibiting serine synthesis reduces S-adenosylmethionine (SAM) levels and decreases histone H3 lysine 27 trimethylation (H3K27me3) modification, thereby alleviating IGF1 inhibition. This promotes IFN-γ-induced M1 polarization via the p38/JAK–STAT1 axis while suppressing IL-4/STAT6-mediated M2 polarization [50]. However, serine simultaneously supports the inflammatory properties of M1 macrophages by activating the mTOR pathway. Serine deprivation inhibits mTOR activity, thereby downregulating NF-κB and NLRP3 inflammasome activation and alleviating inflammatory responses [51].

#### Glutamine

Glutamine metabolism is bidirectionally regulated in DKD; its clinical significance manifests as reduced serum glutamine levels and elevated urinary gamma-glutamyltransferase activity, which serve as early diagnostic markers [52]. Metabolic homeostasis is pivotal in determining the function of glutamine. As a precursor to glutathione (GSH), glutamine, at optimal levels, enhances renal antioxidant enzyme activity (GPx and SOD) while reducing malondialdehyde levels, a lipid peroxidation product. This inhibits ferroptosis and oxidative stress, resulting in decreased expression of renal injury markers [52–54]. However, excess glutamine activates the hexosamine biosynthetic pathway (HBP) under hyperglycemic conditions. It generates uridine diphosphate-N-acetylglucosamine (UDP-GlcNAc) under the action of the rate-limiting enzymes GFPT/GFAT. UDP-GlcNAc subsequently activates PKC and TGF-β signaling. This induces the release of pro-inflammatory cytokines, exacerbating insulin resistance, glomerulosclerosis, and fibrosis [52, 54].

Multiple studies have indicated that glutamine metabolism participates in DKD by regulating phagocyte polarization. The FOXO3A pathway induces high expression of glutamine synthetase (GS) under conditions of glutamine deficiency in the microenvironment, driving M2 polarization [55]. Simultaneously, hormones such as cortisol promote glutamine breakdown by upregulating glutaminase (GLS), thereby enhancing the tricarboxylic acid (TCA) cycle. This further strengthens M2 polarization while suppressing IFN-γ-induced M1 polarization [56]. These findings suggest that targeting glutamine metabolism has therapeutic potential for DKD. Concurrently, animal studies have confirmed that exogenous glutamine supplementation elevates GSH levels, downregulates TNF-α and IL-6 expression, and significantly improves glomerular injury [53]. Intervening in metabolic pathways, such as inhibiting the HBP rate-limiting enzyme GFAT or regulating GS/GLS activity, can reverse metabolic imbalance and alleviate insulin resistance, thereby offering novel therapeutic strategies for DKD [52].

#### Amino Acid Transporters (AATs)

As members of the solute carrier (SLC) superfamily, AATs mediate bidirectional amino acid transport across cell membranes. They are crucial for maintaining cellular nutrient uptake, energy metabolism (e.g., TCA cycle), redox homeostasis (e.g., glutathione synthesis), and mTORC1 signaling activation. In DKD, specific AATs such as SLC38A2 influence mTORC1 signaling and antioxidant capacity by regulating amino acid transport in renal tubular cells. They participate in hyperosmotic stress–induced tubular injury and ferroptosis, promoting disease progression [57]. Hyperglycemia and TGF-β1 signaling significantly suppress the expression of the cystine/glutamate transporter xCT (SLC7A11), thereby reducing cystine uptake and impairing glutathione synthesis. This subsequently decreases GPX4 activity and triggers lipid peroxide accumulation, ultimately causing direct damage to renal tubular cells via ferroptosis [58].

AATs are also involved in macrophage polarization and metabolic reprogramming. Members of the SLC7 family play pivotal roles in this process. A deficiency in SLC7A2-mediated arginine transport simultaneously inhibits iNOS and ARG1 activity, thereby impairing macrophage inflammatory and reparative functions. SLC7A5 activates mTORC1 by transporting leucine, thereby upregulating the expression of pro-inflammatory factors such as IL-6 and TNF-α. Moreover, the cystine–glutamate exchange maintained by SLC7A11 (xCT) influences M2 polarization by regulating glutathione synthesis and oxidative stress, and its dysfunction in DKD amplifies inflammatory injury [59]. Additionally, EAAT2 is highly expressed on the lysosomal membrane of inflammatory macrophages. It maintains the acidic environment of lysosomes and supports macropinocytosis by mediating glutamate/aspartate efflux, thereby activating the V-ATPase proton pump. This process subsequently promotes mTORC1 activation and drives NF-κB signaling and NLRP3 inflammasome assembly, ultimately enhancing IL-1β/TNF-α secretion and promoting macrophage polarization toward the M1 phenotype. This mechanism represents a potential therapeutic target for metabolic inflammation [60].

### Mitochondrial Function-Related Metabolism

#### TCA cycle

Changes in TCA cycle metabolites are key markers of renal dysfunction in DKD. Hyperglycemia drives enhanced glycolysis in the early stages, leading to increased excretion of TCA cycle intermediates (e.g., citrate and malate), reflecting metabolic stress in the kidneys. Mitochondrial dysfunction causes the downregulation of key enzyme expression with a decline in renal function. The levels of urinary TCA cycle metabolites (e.g., methylmalonic acid) decrease significantly and are strongly positively correlated with eGFR, making them sensitive markers of renal impairment [61]. The accumulation of fumarate stabilizes HIF-1α by inhibiting prolyl hydroxylase activity and activating the TGF-β pathway to promote renal fibrosis [62].

**Fig. 7:**
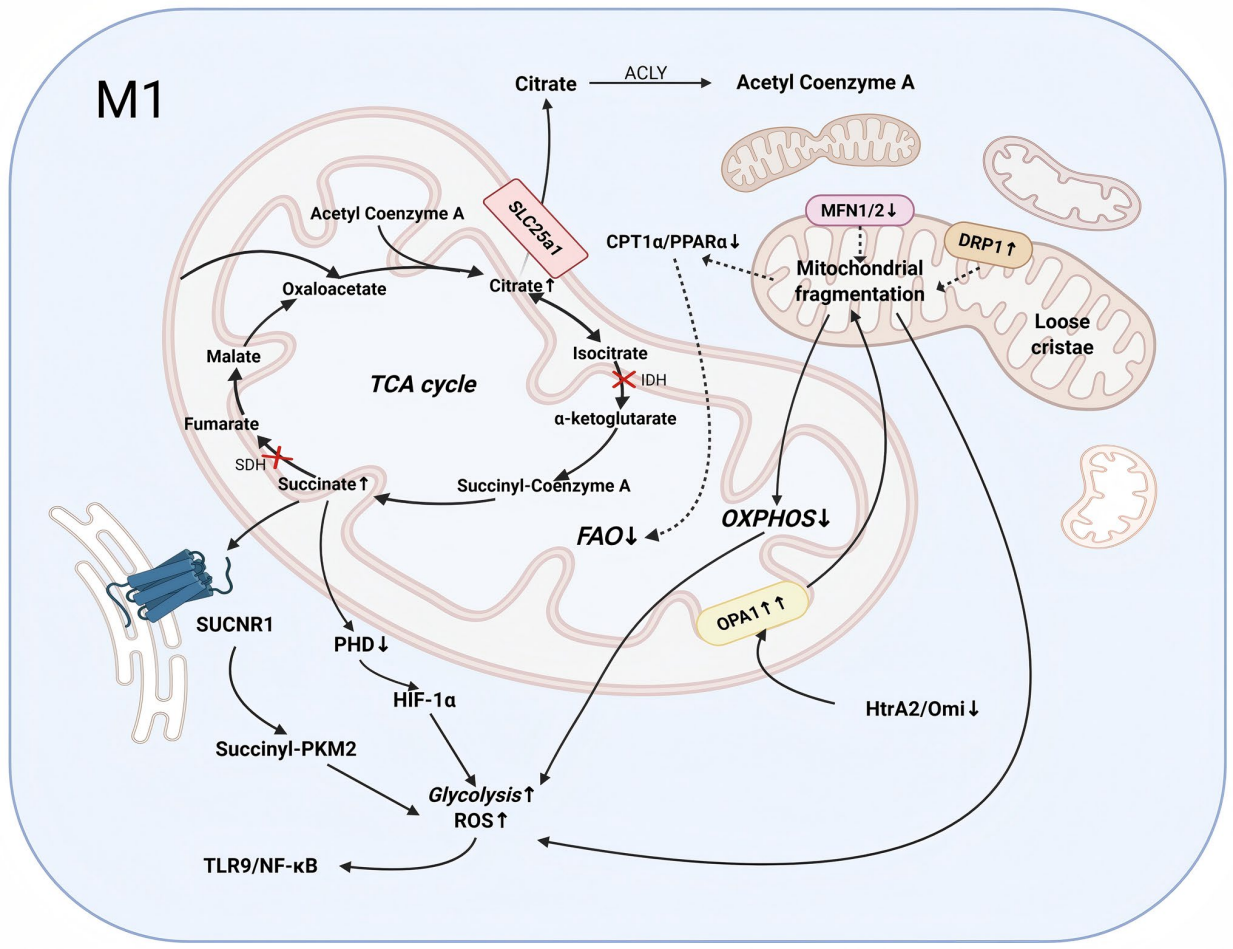
Mitochondrial function-related metabolism in M1 macrophages. *ACLY,* ATP-citrate lyase; *DRP1,* dynamin-related protein 1; *IDH,* isocitrate dehydrogenase; *SDH,* succinate dehydrogenase; *PHD,* prolyl hydroxylase domain; *Succinyl-PKM2,* succinylation of pyruvate kinase M2; *ROS,* reactive oxygen species; *OPA1,* optic atrophy 1; *FAO,* fatty acid oxidation; *MFN1*/*2,* mitofusin 1/2; *OXPHOS,* oxidative phosphorylation

**Fig. 8:**
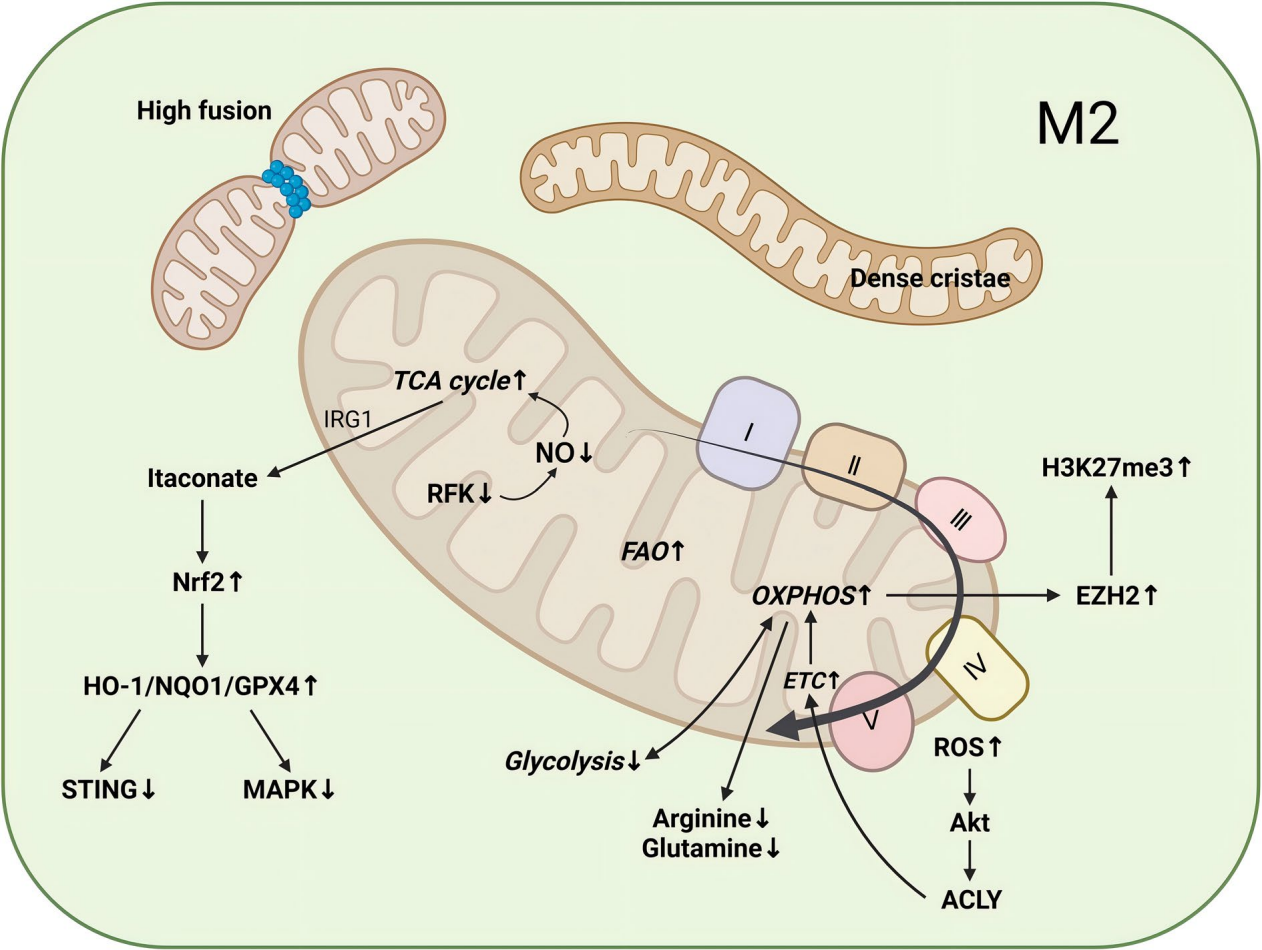
Mitochondrial function-related metabolism in *M2* macrophages. *Nrf2,* nuclear factor erythroid 2-related factor 2; *HO*-1, heme oxygenase 1; *NQO1,* NAD(P)H quinone dehydrogenase 1; *GPX4,* glutathione peroxidase 4; *MAPK,* mitogen-activated protein kinase; *IRG1,* immunoresponsive gene 1; *RFK,* riboflavin kinase; *ETC,* electron transport chain; *EZH2,* enhancer of zeste homolog 2; *H3K27me3,* methylation of lysine 27 on histone H3

The TCA cycle can also regulate macrophage polarization through metabolic remodeling. M1 polarization is characterized by disruption of the TCA cycle at the isocitrate dehydrogenase (IDH) and succinate dehydrogenase (SDH) nodes, leading to the accumulation of citrate and succinate [6] (Fig. 7). Citrate is transported into the cytoplasm via SLC25a1 and broken down by ATP citrate lyase (ACLY) into acetyl-CoA. This drives pro-inflammatory lipid synthesis and histone acetylation, amplifying inflammatory gene expression [6, 63]. Moreover, succinate accumulation activates SUCNR1, which affects PKM2 via succinylation while inhibiting prolyl hydroxylase to stabilize HIF-1α, thereby synergistically inducing glycolysis and ROS release [63, 64]. In contrast, M2 polarization depends on the complete TCA cycle [6] (Fig. 8). Among these compounds, itaconic acid, a metabolite of the TCA cycle, plays a pivotal role. In response to IRG1, its production activates the Nrf2 pathway, upregulating the expression of antioxidant genes such as *HO-1*, *NQO1*, and *GPX4*. This activity inhibits STING and MAPK signaling while promoting M2 polarization [63, 65, 66]. Riboflavin kinase (RFK) deficiency reduces NO levels, thereby inhibiting key TCA cycle enzymes such as pyruvate dehydrogenase (PDH), IDH2, and aconitase (ACO2). This enhances TCA cycle activity, thereby driving M2 polarization [64]. On the basis of this mechanism, targeted interventions such as activating the Nrf2 pathway via iconic acid derivatives or restoring TCA cycle flux and promoting the diversion of arginine metabolism using grossamide [66, 67] offer new directions for improving DKD by targeting macrophage metabolism.

#### Oxidative Phosphorylation

As the core pathway of mitochondrial energy metabolism, oxidative phosphorylation (OXPHOS) drives ATP synthesis through the electron transport chain (ETC) to maintain cellular homeostasis [68]. OXPHOS function has undergone dynamic evolution in DKD. Increased energy demands in renal tubules due to hyperglycemia and sodium‒glucose cotransporter 2 (SGLT2)-mediated glucose reabsorption lead to compensatory enhancement of OXPHOS in early stages [69]. However, hyperglycemia, lipid metabolism disorders, and chronic hypoxia induce dysfunction in ETC complexes I/III/IV with disease progression, leading to a significant decrease in OXPHOS efficiency. This triggers the Warburg effect, resulting in the accumulation of lactate and AGEs [68, 69]. Additionally, human genetic studies have confirmed that mutations in OXPHOS-related genes, such as COQ5 and COX6A1, significantly increase susceptibility to DKD [68].

As a key metabolic regulator of macrophage polarization, enhanced OXPHOS activity drives M2 polarization while suppressing M1 polarization. This process involves a dual mechanism. On the one hand, it inhibits glycolysis and promotes glutamine and arginine metabolism, thereby reducing the release of pro-inflammatory factors such as IL-6 and TNF-α. On the other hand, OXPHOS-driven metabolic reprogramming upregulates the expression of the histone methyltransferase EZH2, thereby increasing the enrichment of the inhibitory epigenetic marker H3K27me3 in the promoter regions of key pro-inflammatory genes, thus suppressing M1 polarization [70]. At the molecular level, during enhanced OXPHOS, the activity of mitochondrial complexes I–V increases. This activates ACLY via the ROS/Akt pathway to stabilize cardiolipin and maintain ETC function [71]. The NLRX1 deficiency-induced enhancement of OXPHOS directly drives M2 polarization in the renal microenvironment, thereby inducing the expression of M2 markers such as Mgl1 and Mrc1 as well as the secretion of TGF-β [72]. Similarly, 15-lipoxygenase deficiency promotes macrophage polarization toward M2c cells by inhibiting glycolysis and enhancing OXPHOS. This reduces M1 macrophage infiltration and downregulates cytokines such as TNF-α, thereby alleviating inflammation and fibrosis [73].

On the basis of the aforementioned findings, targeting OXPHOS is expected to emerge as a novel therapeutic strategy for DKD. SGLT2 inhibitors and glucagon-like peptide-1 receptor agonists (GLP-1RAs) may slow disease progression by enhancing mitochondrial biogenesis and OXPHOS [69]. Additionally, experiments with the mitochondrial inhibitor CPI-613 demonstrated that restoring PINK1–MFN2 axis function through MFN2 or PINK1 overexpression effectively reversed OXPHOS inhibition and promoted the shift of macrophages from the M1 to M2 phenotype, thereby providing a targeted metabolic therapeutic approach for DKD [74].

#### Mitochondrial Dynamics

Mitochondrial dynamics is a key mechanism that regulates mitochondrial morphology, number, distribution, and function in eukaryotic cells through the continuous processes of fission, fusion, autophagy, and transport. This process is centrally regulated by proteins such as the fission-promoting protein DRP1 and the fusion-promoting proteins MFN1/2 and OPA1. Imbalances in these processes can lead to metabolic disorders, ROS accumulation, and cellular dysfunction, resulting in various diseases [75]. In DKD, pathological factors such as hyperglycemia and lipotoxicity can induce mitochondrial dysregulation. This primarily manifested as DRP1-mediated excessive fission and downregulation of MFN1/2 and OPA1 expression, leading to mitochondrial fragmentation. Concurrently, impaired mitochondrial autophagy mediated by the PINK1/Parkin pathway further triggers mtROS bursts and energy metabolism dysfunction. This process promotes apoptosis and fibrosis through signaling pathways such as JNK–p53 pathway [76–78].

Macrophages with varying phenotypes exhibit distinct mitochondrial morphological characteristics. M1 macrophages exhibit mitochondrial fragmentation and loose cristae. This morphology suppresses OXPHOS, drives glycolytic dominance, and promotes ROS production to enhance inflammatory responses. In contrast, M2 macrophages possess a highly fused mitochondrial network with dense cristae, which supports efficient OXPHOS and FAO, thereby maintaining their anti-inflammatory and reparative functions [79]. Disorders of mitochondrial dynamics can regulate macrophage polarization through multiple pathways. For instance, reduced HtrA2/Omi promotes excessive OPA1 accumulation. This accelerates mitochondrial fragmentation, inhibits OXPHOS, and enhances the release of glycolysis and ROS. This further activates the TLR9/NF-κB pathway, thereby driving M1 polarization [80]. In DKD, macrophages predominantly exhibit anti-inflammatory functions in the context of mitochondrial dynamics disorders. Specifically, Mitochondrial dysfunction—driven by diminished MFN1 and MFN2 expression and elevated DRP1 activity—suppresses FAO through coordinated downregulation of PPARα and CPT1A, resulting in intracellular lipid accumulation (lipotoxicity) and subsequent release of inflammatory cytokines, including TNF-α and IL-6. This promotes M1 polarization while inhibiting M2 polarization [81]. Simultaneously, the activation of the Hippo pathway and inactivation of YAP1 in renal tubular cells disrupt mitochondrial quality control, leading to the secretion of chemokines such as CXCL1. These chemokines recruit macrophages via a paracrine mechanism, promoting pro-inflammatory M1 polarization while suppressing M2 polarization, thus aggravating renal injury [82]. These findings indicate that mitochondrial dynamics in macrophages play a pivotal role in the pathogenesis of DKD and may contribute to maintaining M1 polarization.

## Targeting Metabolic Reprogramming to Regulate Macrophage Polarization to Alleviate DKD

### Glucose Metabolism

#### TAK1/TAB1 Complex Inhibitor

Transforming growth factor beta–activated kinase 1–binding protein 1 (TAB1) is a key binding protein for transforming growth factor beta–activated kinase 1 (TAK1). It forms a complex with TAK1 through constitutive binding to its carboxy-terminal domain. This complex promotes TAK1 autophosphorylation and regulates its kinase activity even in the resting state, making it a critical regulator of both the NF-κB and MAPK signaling pathways [83]. High-glucose environments in DKD activate TAB1 in macrophages, which upregulates key glycolytic enzymes via the NF-κB/HIF-1α axis. This drives glycolysis-dependent M1 polarization and subsequent renal injury. Inhibiting TAB1 effectively blocks this pathway, yielding therapeutic effects [84].

Multiple drugs can influence macrophage polarization by inhibiting the formation of the TAK1/TAB1 complex. The novel triptolide derivative ZT01 directly binds to TAK1, inhibiting its phosphorylation and kinase activity thereby blocking the downstream MKK4/JNK pathway (Table 1). It specifically suppresses M1 polarization without affecting M2 marker expression and indirectly promotes an anti-inflammatory microenvironment. Currently evaluated as a therapeutic candidate for inflammatory diseases such as sepsis, it significantly reduces tissue damage in LPS- and CLP-induced mouse sepsis models [85]. Future studies may explore its potential therapeutic value in DKD.

**Table 1:** Potential drug candidates targeting metabolic reprogramming to regulate macrophage polarization for alleviating DKD [34, 45, 67, 85–105]

Targeted energy metabolic pathways	Potential drug candidates	Classification	Targets	Macrophage polarization	References
Glucose metabolism	ZT01	Chemical drug	TAK1/TAB1	M1↓	[85]
	α-Momordicin	Active ingredient	TAK1	M1↓	[86]
	Quercetin	Active ingredient	Src/Syk kinases	M1↓	[87]
	Shenhua tablet	Traditional Chinese medicine formulas	HIF-1α/PKM2	M1↓, M2↑	[88, 89]
	Sulforaphane	Active ingredient	PKM2	M1↓	[90]
	Lycium barbarum polysaccharides	Active ingredient	PKM2	M1↓, M2↑	[91]
	Cichoric acid	Active ingredient	HIF-1α	M1↓, M2↑	[92]
Lipid metabolism	Esculetin	Active ingredient	Carnitine palmitoyltransferase 1A	M2↑	[93, 94]
	Tectorigenin	Active ingredient	AdipoR1/2	M2↑	[95]
	Qinghao-biejia herb pair	Traditional Chinese medicine formulas	ABCA1/ABCG1	M1↓, M2↑	[96]
	Atorvastatin	Chemical drug	Mevalonate pathway	M1↓, M2↑	[34, 97]
	Fluvastatin	Chemical drug	Mevalonate pathway	M1↓, M2↑	[97, 98]
Amino acid metabolism	1,7-Dihydroxy-3,4-dimethoxyoxoanthracenone	Active ingredient	Arginase-1	M1↓	[99]
	Jianpi-Yishen formula	Traditional Chinese medicine formulas	Betaine	M2↑	[45]
Mitochondrial function related metabolism	Grossamide	Active ingredient	IDH1, SDH	M1↓, M2↑	[100]
	Songorine	Active ingredient	Glycolytic enzymes	M1↓, M2↑	[101]
	Paeoniflorin	Active ingredient	Krippel-like transcription factor 4	M1↓, M2↑	[102]
	Luteolin	Active ingredient	DRP1, MFN1/2, OPA1	M2↑	[103]
	Muscone	Active ingredient	DRP1	M2↑	[67]
	Rapamycin	Chemical drug	mTOR	M1↓	[104, 105]

α-Momordicin (α-MMC) derived from bitter melon inhibits TAK1 phosphorylation in a dose-dependent manner, thereby blocking the NF-κB and MAPK pathways downstream of TLR4. This effectively reduced the release of pro-inflammatory factors by M1 macrophages and alleviated inflammatory responses and tissue damage in an LPS-induced acute pneumonia mouse model without interfering with the anti-inflammatory functions of M2 macrophages, highlighting the potential of M2 macrophages for treating inflammatory diseases [89].

Quercetin, a flavonoid widely present in fruits and vegetables, inhibits the formation of the TLR4/MyD88/PI3K complex by suppressing Src/Syk kinases. This subsequently blocks the IRAK1–TRAF6–TAK1/TAB1 signaling axis and the downstream NF-κB and MAPK pathways, ultimately downregulating the expression of M1-associated inflammatory mediators. It has been shown to have significant anti-inflammatory effects in LPS-induced RAW 264.7 cells and in mouse inflammation models [92].

#### HIF-1α/PKM2 Inhibitor

Multiple studies have demonstrated that drugs can induce M2 polarization in macrophages by inhibiting HIF-1α/PKM2 signaling and alleviating inflammatory injury.

Shenhua tablet (SHT) downregulates the expression of the key glycolytic enzymes HK1 and LDHA by inhibiting the HIF-1α/PKM2 pathway, thereby reducing glycolytic activity in macrophages. This metabolic reprogramming promotes the transformation of macrophages from the M1 phenotype to the M2 phenotype and reduces the release of inflammatory mediators such as TNF-α and IL-1β, thereby suppressing immune-inflammatory responses [86]. Early clinical studies further suggest that the efficacy of SHT in reducing proteinuria is comparable to that of angiotensin-converting enzyme inhibitors, with favorable safety profiles, providing preliminary evidence for its use as an adjunctive therapy in DKD [88].

Sulforaphane (SfN) is an aliphatic isothiocyanate that inhibits the glutathionylation of PKM2, maintaining its highly active tetrameric conformation. This prevents PKM2 from entering the nucleus and interacting with HIF-1α, thereby reducing HIF-1α stability and inhibiting Stat3 phosphorylation. Ultimately, this activity blocks M1 polarization and suppresses the expression of pro-inflammatory cytokines such as IL-1β and has significant anti-inflammatory effects in LPS-induced macrophage inflammation models [90], highlighting its potential for treating inflammatory diseases.

Lycium barbarum polysaccharides (LBPs) are the primary bioactive components of Chinese wolfberry. They reversed LPS-induced M1 polarization by promoting PKM2 ubiquitination and degradation through upregulation of ubiquitin ligase activity, thereby blocking PKM2–HIF-1α interactions, inhibiting glycolysis and lactate production, reducing CD86 and pro-inflammatory factor expression, and restoring M2-associated factor expression. In a RAW264.7 macrophage model, they demonstrated significant anti-inflammatory effects [91].

Cichoric acid (CA) is a natural organic compound that is primarily isolated from Asteraceae plants and is widely used in health foods and pharmaceuticals. CA downregulates HIF-1α expression by inhibiting SDH activity, thereby reducing succinate accumulation and ROS production. This suppresses PKM2 expression and lactate generation, blocking M1 polarization of macrophages. In an LPS-induced acute kidney injury mouse model, CA pretreatment demonstrated significant renal protective effects [87].

### Lipid Metabolism

#### Fatty Acids

Esculetin (ELT) activates β-oxidation metabolism in macrophages by upregulating the expression of carnitine palmitoyltransferase 1A. This reprogramming directly drives M2 polarization, manifested by significantly increased expression of M2 markers such as CD206 and Arg-1, as well as anti-inflammatory cytokines such as IL-4 and IL-10 [94]. In a preclinical model of diabetes complicated by acute kidney injury, ELT intervention markedly improved renal dysfunction and effectively reduced renal tissue pathology scores, demonstrating its therapeutic potential in DKD [97].

Tectorigenin is an isoflavone compound extracted from the rhizomes of iris plants. It activates the AdipoR1/2-AMPKα-PPARα pathway, thus enhancing FAO while inhibiting lipid synthesis. This process reduces renal macrophage infiltration in db/db mice with DKD, delays glomerular dysfunction and fibrosis by suppressing NF-κB activation, downregulating M1-associated pro-inflammatory cytokines, and simultaneously increasing M2-associated anti-inflammatory cytokine expression [98].

#### Cholesterol

Qinghao-biejia herb pair (QB) can upregulate ABCA1/ABCG1 by activating the liver X receptor α (LXR-α) signaling pathway, thereby promoting cholesterol efflux from macrophages and reducing lipid accumulation. This drives their polarization from the M1 to M2 phenotype, manifested by decreased pro-inflammatory cytokines and increased IL-10 and Ym-1 levels in a preclinical model of systemic lupus erythematosus with atherosclerosis [95].

Statins achieve systemic anti-inflammatory effects by reducing LDL cholesterol and its aggregation in lipid rafts, inhibiting lipid raft-dependent pro-inflammatory cytokine expression, directly promoting M1-to-M2 transformation, and decreasing the proportion of tissue M1 macrophages [34]. Specifically, atorvastatin and fluvastatin inhibit cholesterol synthesis by suppressing the mevalonate pathway, thereby further regulating macrophage M1 polarization and alleviating tissue injury [93, 96].

### Amino Acid Metabolism

1,7-Dihydroxy-3,4-dimethoxyoxoanthracenone (XAN), derived from the South China medicinal herb Cynanchum wilfordii, promotes the transformation of arginine into polyamines by increasing the expression of key enzymes such as Arg-1. Polyamines scavenge ROS, mitigate mitochondrial damage, and inhibit NLRP3 inflammasome activation, thereby reducing pro-inflammatory cytokine release and suppressing M1 polarization. Studies have revealed that in LPS/IFN-γ-induced macrophage inflammation models, XAN exerts significant anti-inflammatory effects. It effectively suppresses the release of key pro-inflammatory factors while promoting the expression of anti-inflammatory mediators. Moreover, it alleviates inflammatory responses by regulating macrophage polarization balance, providing experimental evidence for the therapeutic potential of XAN in inflammatory diseases [99].

The core mechanism of the traditional Chinese medicine Jianpi-Yishen formula involves significantly elevated betaine levels in renal tissue. This activates glycine, serine, and threonine metabolic pathways, promoting M2 polarization in macrophages. M2 macrophages suppress local renal inflammatory responses by secreting anti-inflammatory factors, thereby slowing the progression of CKD. In a mouse model of CKD, treatment with Jianpi-Yishen formula significantly reduced serum creatinine and blood urea nitrogen levels while effectively alleviating renal inflammatory damage, demonstrating its therapeutic potential for DKD [45].

### Mitochondrial Function-Related Metabolism

#### TCA Cycle

Grossamide (GSE) upregulates arginase and inhibits iNOS, thereby alleviating the suppression of OXPHOS by NO. Moreover, it restored the activity of key TCA cycle enzymes such as IDH1 and SDH, reversing metabolic disruption and shifting energy metabolism from glycolysis to OXPHOS. This metabolic reprogramming reduces pro-inflammatory cytokine secretion by inhibiting the TLR4/NF-κB pathway, promoting the transformation of macrophages from the M1 to the M2 phenotype, and holds therapeutic potential for DKD [67].

#### OXPHOS

Songorine is a diterpene alkaloid extracted from Aconitum plants. It inhibits the expression of key glycolytic enzymes while enhancing mitochondrial OXPHOS. This metabolic shift promotes M1-to-M2 polarization in macrophages and reduces ROS production by increasing the NAD⁺/NADH ratio, ultimately inhibiting the release of inflammatory cytokines such as IL-1β and IL-6. These findings confirm the pivotal role of the OXPHOS pathway in regulating the immune microenvironment [101]. Therefore, further research is needed to elucidate the therapeutic effects of songorine on DKD.

#### Mitochondrial Dynamics

Paeoniflorin, a monoterpenoid glycoside extracted from peony roots, activates mitochondrial autophagy to mediate PINK1/Parkin- and Bnip3-dependent clearance of damaged mitochondria, thereby upregulating the expression of Krippel-like transcription factor 4 (KLF4) and driving macrophage polarization in CKD mouse models during preclinical studies from M1 to M2 and alleviating renal inflammation [102]. Therefore, paeoniflorin appears to alleviate DKD by modulating macrophage polarization.

Luteolin (LUT) is a natural flavonoid compound found in various plants. It improves the mitochondrial fusion–fission balance by downregulating DRP1 expression and upregulating MFN1/2 and OPA1 expression, thereby restoring mitochondrial function. This promotes M2 polarization and suppresses inflammatory responses in rat models used in preclinical research on periodontal disease [100]. Therefore, LUT should be considered for the clinical treatment of DKD in the future.

Muscone is the primary aromatic component of natural musk and serves as a key ingredient in numerous traditional Chinese patent medicines. By inhibiting DRP1 activation and its translocation to mitochondria, it mitigates excessive mitochondrial fission, maintains membrane potential, and reduces ROS production. This subsequently suppresses the NLRP3 and NF-κB pathways, promoting M2 polarization in macrophages. In preclinical studies, muscone demonstrated significant therapeutic potential, effectively improving motor function while reducing inflammatory responses and neuronal damage in a rat cervical spinal cord injury model. In vitro experiments further confirmed its ability to suppress inflammatory cytokine expression and exert neuroprotective effects, substantiating its therapeutic value for inflammatory diseases such as DKD [104].

Research has revealed that TREM-1 activation promotes DRP1 Ser616 phosphorylation and MTFP1/PGAM5 expression through an mTOR-dependent pathway, leading to excessive mitochondrial fission, disrupted membrane potential, and abnormal mitochondrial autophagy. This ultimately triggers RIPK3/MLKL-mediated necrotic apoptosis in macrophages and the release of pro-inflammatory factors such as TNF-α and IL-1β. The drug rapamycin, an immunosuppressant that blocks mTOR activity, significantly inhibits DRP1-dependent mitochondrial fission, restores mitochondrial homeostasis, reduces necrotic apoptosis, and consequently alleviates inflammatory responses [103]. In preclinical studies of DKD, rapamycin has demonstrated significant renoprotective effects in animal models. This conclusion is supported by intervention studies in diabetic mice, which indicate that the drug effectively reduces glomerular hypertrophy, proteinuria, and renal cell injury, independent of its blood glucose-regulating effects [105]. Therefore, rapamycin may be used to treat DKD in the future.

## DKD Therapeutic Agents and Their Effects on Macrophage Polarization and Metabolism

In accordance with the “KDIGO 2022 Clinical Practice Guideline for the Management of Chronic Kidney Disease and Diabetes”, medications commonly recommended for patients with DKD include SGLT2 inhibitors, renin–angiotensin–aldosterone system (RAAS) inhibitors, GLP-1RAs, and nonsteroidal mineralocorticoid receptor antagonists (MRAs) [106]. These agents have been shown to exert therapeutic effects on DKD by modulating macrophage polarization through mechanisms such as alterations in metabolic pathways.

Research has indicated that SGLT2 inhibitors, such as canagliflozin, suppress mTORC1 by activating AMPK while simultaneously inhibiting the PI3K/Akt and HIF-1α signaling pathways, thereby reducing glycolysis-related expression. The accumulation of its metabolite itaconic acid activates the Nrf2 pathway and inhibits the expression of GAPDH, promoting the polarization of macrophages from the M1 phenotype to the M2 phenotype [107]. Different types of RAAS inhibitors reverse the process by which angiotensin Ⅱ (Ang Ⅱ) promotes M1 polarization through pathways such as NF-κB activation via AT1R, which acts through distinct mechanisms [108]. Angiotensin-converting enzyme inhibitors (ACEIs), such as enalapril, weaken the drive for M1 polarization at its source by reducing Ang Ⅱ production. Angiotensin receptor blockers (ARBs), such as losartan, directly block AT1R to inhibit downstream inflammatory signaling. Notably, certain ARBs, such as telmisartan, exert effects independent of AT1R blockade—directly promoting M2 polarization by activating PPARγ. These synergistic mechanisms significantly reduce M1 factor release while increasing M2 factor secretion, thereby alleviating renal inflammatory infiltration, proteinuria, and renal fibrosis [109]. GLP-1RAs activate the GLP-1R–PKA signaling axis to remodel mitochondrial function. By enhancing FAO, upregulating ETC activity, and inhibiting glycolysis, they reduce oxidative stress and lipid accumulation, thereby indirectly suppressing NLRP3 inflammasome activation in macrophages [110]. Moreover, it directly regulates immune cell metabolism and signaling pathways; on the one hand, it inhibits hyperglycemia-driven glycolysis, downregulates ADAM17/iRhom2 expression to reduce TNF-α release, and protects podocytes [111]; on the other hand, it regulates the STAT signaling pathway by inhibiting STAT1 phosphorylation to reduce M1 polarization while enhancing STAT3/STAT6 phosphorylation to promote M2 polarization [112]. These interconnected mechanisms collectively promote M2 polarization, ultimately alleviating renal inflammation. Selective MRAs, such as finerenone, exert renoprotective effects through both metabolic and immune mechanisms by blocking abnormally activated MR signaling. At the mitochondrial level, they activate the PI3K/Akt/eNOS pathway, promoting mitochondrial biogenesis and alleviating oxidative stress [113]. At the immunoregulatory level, they antagonize MR in macrophages and downregulate the expression of the G protein subunit Gnxi2, thereby suppressing excessive activation of the complement C5a–C5aR1 axis, reducing inflammatory chemokine release, and driving macrophage polarization from the M1 phenotype to the M2 phenotype [114]. These dual pathways collectively mitigate inflammation and tissue fibrosis in DKD.

## Conclusions

DKD is among the most common and severe microvascular complications of diabetes, with approximately 40% of patients with T2D and 30% of patients with T1D ultimately progressing to DKD. Its typical pathological features include persistent proteinuria, a decreased glomerular filtration rate, and thickening of the glomerular basement membrane. Current clinical strategies for controlling blood glucose and lipid levels have limited efficacy, underscoring the urgent need to develop new therapeutic approaches.

Macrophages play a dual role in the progression of DKD. Renal macrophages primarily consist of resident macrophages and those derived from monocytes and exhibit distinct differences in origin, metabolic characteristics, and function. Macrophages can be polarized into pro-inflammatory M1 or anti-inflammatory M2 phenotypes based on microenvironmental signals. M1 macrophages exacerbate renal inflammation and tissue damage by releasing inflammatory mediators such as TNF-α, IL-1β, IL-6, and ROS. In contrast, M2 macrophages highly express surface markers such as Arg-1 and CD206 while secreting cytokines such as IL-10 and TGF-β, thereby promoting tissue repair and mitigating fibrosis progression. However, M2 macrophage function is often suppressed in high-glucose environments, leading to inadequate control of inflammation and even further progression of fibrosis.

Metabolic reprogramming is the core regulatory mechanism of macrophage polarization. This review systematically summarizes the roles of glucose metabolism, lipid metabolism, amino acid metabolism, and mitochondrial function in macrophage polarization. Specifically, enhanced glycolysis and activation of the HIF-1α/PKM2 signaling pathway primarily promote M1 polarization, whereas increased FAO facilitates M2 polarization. Arginine metabolism regulates M1/M2 phenotype switching via the balance between iNOS and Arg1. Mitochondrial integrity and OXPHOS activity form the essential metabolic foundation for M2 polarization. Although fully elucidating the absolute temporal sequence and hierarchical relationships of metabolic reprogramming events in DKD remains challenging, an evidence-based inferential framework can be proposed. Enhanced glycolysis and HIF-1α signaling activated by a high-glucose environment represent early events that initiate immune-metabolic dysregulation in the kidney. Mitochondrial dysfunction, as a direct consequence of impaired OXPHOS, may serve as the fundamental upstream driver propelling this compensatory glycolytic enhancement. In this context, sustained activation of the HIF-1α/glycolysis axis constitutes a core driving and amplifying node. Through mechanisms such as stabilizing HIF-1α, promoting glycolysis, and the accumulation of lactate and succinate, it not only exacerbates mitochondrial dysfunction but also forms a self-reinforcing positive feedback network with processes such as lipid metabolism disorders. This network is dominant and sustains M1 macrophage polarization. Therefore, simultaneously targeting improvements in mitochondrial function and intervening in the HIF-1α/glycolysis axis is considered a highly promising strategy for overcoming this vicious cycle.

On the basis of these mechanisms, targeting metabolic reprogramming to modulate macrophage polarization has emerged as a novel therapeutic strategy for DKD. Various natural compounds (e.g., quercetin, lithospermin, and sulforaphane) and synthetic drugs (e.g., SGLT2 inhibitors and statins) effectively induce M2 polarization and suppress M1 polarization by inhibiting glycolysis, promoting FAO, regulating amino acid metabolism, or enhancing mitochondrial biogenesis and OXPHOS. These agents have demonstrated promising potential in reducing renal inflammation and fibrosis in preclinical studies, offering adjunctive therapeutic prospects for DKD. While preclinical results are promising, translation to confirmed human benefits requires future clinical trials. Additionally, conventional drugs such as SGLT2 inhibitors have been shown to drive the polarization of macrophages from M1 to M2 through intervention in specific metabolic and immune signaling pathways, thereby alleviating renal inflammation, cellular injury, and the progression of fibrosis. However, macrophage polarization exhibits distinct spatiotemporal dynamics and microenvironmental dependence. Current in-depth metabolic studies largely focus on the broad M1/M2 classification, with an understanding of whether distinct M2 subtypes possess unique metabolic signatures—such as whether the M2a subtype relies more heavily on specific metabolic pathways—still in its infancy. Therefore, precisely deciphering the mechanisms and targets of metabolic reprogramming in each M2 subtype undoubtedly represents one of the most challenging yet promising frontier directions in future DKD immunometabolic research. Elucidating this mechanism not only offers a novel explanation for DKD pathogenesis but also holds promise for establishing a theoretical foundation for developing precise metabolic interventions targeting M1/M2 macrophage subtypes. These findings will advance the clinical translation of macrophage polarization regulation through targeted metabolic reprogramming, thereby optimizing therapeutic strategies for DKD.

## Data Availability

Not applicable.
